# Marine Natural Products from Microalgae: An -Omics Overview

**DOI:** 10.3390/md17050269

**Published:** 2019-05-07

**Authors:** Chiara Lauritano, Maria Immacolata Ferrante, Alessandra Rogato

**Affiliations:** 1 Marine Biotechnology Department, Stazione Zoologica Anton Dohrn, Villa Comunale, 80121 Naples, Italy; chiara.lauritano@szn.it; 2 Integrative Marine Ecology Department, Stazione Zoologica Anton Dohrn, Villa Comunale, 80121 Naples, Italy; 3 Institute of Biosciences and BioResources, CNR, Via P. Castellino 111, 80131 Naples, Italy

**Keywords:** marine microalgae, marine natural products (MNPs), bioactive compounds, blue biotechnology, genomics, transcriptomics, proteomics, metabolomics, genetic engineering, bioinformatics

## Abstract

Over the last decade, genome sequences and other -omics datasets have been produced for a wide range of microalgae, and several others are on the way. Marine microalgae possess distinct and unique metabolic pathways, and can potentially produce specific secondary metabolites with biological activity (e.g., antipredator, allelopathic, antiproliferative, cytotoxic, anticancer, photoprotective, as well as anti-infective and antifouling activities). Because microalgae are very diverse, and adapted to a broad variety of environmental conditions, the chances to find novel and unexplored bioactive metabolites with properties of interest for biotechnological and biomedical applications are high. This review presents a comprehensive overview of the current efforts and of the available solutions to produce, explore and exploit -omics datasets, with the aim of identifying species and strains with the highest potential for the identification of novel marine natural products. In addition, funding efforts for the implementation of marine microalgal -omics resources and future perspectives are presented as well.

## 1. Introduction

The advent of the -omics era, and the development of the related technologies for the acquisition and analysis of the amount of big datasets available, has revolutionized biological research. This holistic approach promises to discover and understand new patterns, and has diverse and important applications in the field of biotechnology [1]. In the last decade, growing public and private interest and investment in the marine biotechnology (or blue biotechnology) have increased the opportunity to generate information, and to collect huge amounts of data to understand different cellular processes and biological phenomena. Blue biotechnology makes use as well of multi-omics methodologies (such as genomics, transcriptomics, proteomics, metabolomics, metagenomics and metatranscriptomics) for the production and analysis of massive biological data. One of the most promising outcomes of the blue biotechnology research is the discovery of marine natural products (MNPs), defined as bioactive compounds derived from marine organisms [2,3,4,5,6]. 

The number of potential MNPs isolated now exceeds 28,000, with hundreds of new compounds being discovered every year [7], with 1490 of these new compounds recorded in 2017 alone [8]. Microalgae biotechnology is a growing domain in this field, and recently there is a renewed interest in exploring and exploiting microalgal properties for the identification and characterization of new MNPs [2,3]. Microalgae are known to be excellent sources of pigments, lipids, including omega-3 fatty acids, vitamins, toxins and other chemicals [9,10].

Recent studies have shown that various microalgae may have antiproliferative and anticancer activities [11]. In their review, Martinez Andrade et al. [11] reported microalgae which have shown activities against human cancer cell lines, such as the diatoms *Phaeodactylum tricornutum*, *Skeletonema marinoi* and *Chaetoceros calcitrans,* and the dinoflagellates *Ostreopsis ovata* and *Amphidinium operculatum*. Driven by the increasing rate of antibiotic-resistant bacteria and infections, microalgae have been also recently studied to identify new anti-infective compounds [12]. For example, Lauritano et al. [12] found for the first time that extracts of two diatoms, *Skeletonema costatum* and *Chaetoceros pseudocurvisetus*, had antituberculosis activity. Most microalgal bioactive compounds can be ingested directly, as in the case of omega-3 fatty acids or astaxanthin. Conversely, other compounds, such as toxins, can be used as lead compounds to be “improved” (e.g., structure modification), in order to reduce toxicity, increase specificity, improve duration of action and compound stability and/or reduce the costs of synthesis production [13]. 

The selection of microalgal strains for possible biotechnological applications is generally done depending upon key characteristics (e.g., easy to culture and fast growing strains). In addition, various factors need to be taken into account when culturing a microalga, such as the proper conditions (e.g., light, temperature, air bubbling, nutrients, salinity and pH) and the possible presence of associated bacteria. If the strain is not axenic, the algal bioactivity may be due to the bacterial presence. Isolation and cultivation are still problematic for a number of species observed in natural samples, or detected in meta-genomics datasets, however for some of the most widely used microalgal species, mass cultivation is easier compared to other marine organisms, especially macro-organisms. They generally have short generation times (doubling time of 5–8 h for some species), can be mass cultivated in photobioreactors, allowing them to overcome problems associated with the over-utilization of marine resources and the use of destructive collection practices. 

Moreover, for a significant number of species, protocols for genetic manipulation are available to improve their potential as sources of active compounds. 

The most common pipeline for drug discovery from microalgae includes their cultivation in small or large volumes (e.g., by using photobioreactors), their pellet concentration (by centrifugation or filtration), chemical extraction of the microalgal pellets, which can be performed with various protocols in order to find the metabolites of interest, e.g., [12], and screening of the extracts for different bioactivities (e.g., antioxidant, anti-inflammatory, antimicrobial and anticancer testing). Bioactivity-guided fractionation allows to identify the active fraction and, likely, the active compound. The chance to obtain a continuous source of MNPs from marine microalgae, generally more amenable to culturing compared to macro-organisms, may be able to meet the challenging demands of the food, nutraceutical and cosmaceutical market. Indeed, they represent a renewable and still poorly explored resource for drug discovery.

**Figure 1:**
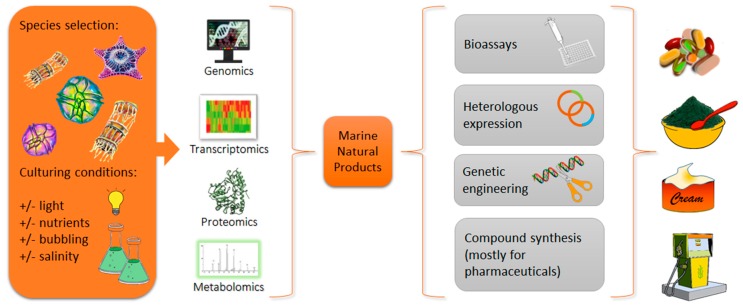
Schematic representation of drug discovery from microalgae with -omics approaches (i.e., genomics, transcriptomics, proteomics and metabolomics) in order to identify marine natural products (MNPs). Once the compound of interest is identified, bioactivity screening, heterologous expression, genetic engineering and/or chemical synthesis can follow. This will allow us to characterize the activity and produce the desired bioproducts with pharmaceutical, nutraceutical, cosmeceutical and biofuel production applications.

In addition to the pipeline for compound discovery just described, other approaches have been used for microalgae, such as identifying enzymatic pathways responsible for the synthesis of bioactives (via genomics and transcriptomics analyses), or by looking at the entire microalgal content of proteins and metabolites (by proteomics and metabolomics investigations). As shown in Figure 1, microalgae are first cultivated in control or stressful conditions e.g., modifying light exposure, salinity or nutrient concentration, to trigger production of the widest range of metabolites, or to enhance production of selected ones (as performed by [14,15]). When the culturing parameters are modified (a strategy known as “One Strain—MAny Compounds” (OSMAC) [6]), the same species may produce different metabolites, and have diverse bioactivities [16]. Obtained microalgal cultures can then be used to extract RNA, DNA and proteins, which can be massively sequenced and analyzed with bioinformatics tools in order to perform drug discovery with in silico approaches. 

In silico identification is then confirmed/implemented by bioactivity screening, heterologous expression, genetic engineering and/or chemical synthesis, in order to produce higher amounts of the metabolite of interest, and meet the industrial demand (Figure 1). This review will focus specifically on -omics approaches applied to marine microalgae to obtain products for different market sectors (e.g., nutraceuticals, pharmaceuticals, cosmeceuticals), and for biofuel production. Integrating and cross-linking by using several bioinformatics tools will facilitate the detection of known MNPs, as well as the discovery of new compounds. We also briefly review the recent research efforts in applying genetic engineering methods to marine microalgae to develop organisms optimized for the high productivity of bioactive compounds of interest. Finally, we report an excursus of several European FP7 and H2020-funded projects, under the topic “Blue growth”, with the common goal of achieving a more environmentally friendly approach to drug discovery.

## 2. -Omics Datasets Available for Marine Microalgae

### 2.1. Genomics

Over the last decade, marine biologists have been applying genomics approaches at both organism and ecosystem levels, and the amount of genomic resources for marine organisms is now becoming significant. One of the main limitations for the use of these resources is the lack of comprehensive websites where all data are available. Marine microalgal genomes (Table 1) can be found in several databases, such as the Joint Genome Institute (JGI) Genome Portal, which provides unified access to JGI genomic data that can be searched, downloaded and explored with several analytical tools [17], or Pico-Plaza, another access point for algal comparative genomics centralizing genomic data, produced by different genome sequencing initiatives [18]. BioCyc, a microbial genome web portal that combines thousands of genomes with information imported from other databases, is another website where some microalgal genomes can be browsed [19]. A limited number of well-refined genomes for microalgae are available in Ensembl Protists (Table 2), a section of the Ensembl genome browser originally developed for vertebrates by the European Bioinformatics Institute (EBI) and the Wellcome Trust Sanger Institute, which is used for the annotation, analysis and display of genomes [20]. Considering only diatoms, one of the major groups of marine unicellular algae, Ensembl Protists contains the genome of four species, *P. tricornutum*, *Thalassiosira pseudonana*, *Thalassiosira oceanica* and *Fragilariopsis cylindrus.*

However, there are other published diatom genomes [21,22,23,24], and one publicly available, unpublished genome (*Pseudo-nitzschia multiseries*, JGI website), which are more or less easily accessible, with resources scattered in different places. Similarly, genomes for other marine microalgae are often available in different databases (as summarized in Table 2).

As for other non-model organisms, this fragmentation represents a limitation for the development of tools that allow the comparison of different genomic data to infer information about gene families and function. A few sites have been implemented (http://www.gene2function.de/, tools in Pico-Plaza), but it will still take time to develop resources comparable to those available in other fields (such as TreeFam [25], PlantGDB, http://www.plantgdb.org/site/, antiSMASH, [26]). 

Genome sequences can be instrumental in the reconstruction of metabolic pathways. The presence of expanded gene families in given species has been at times linked to enhanced metabolic abilities. A detailed annotation of enzymes linked to carbohydrate and lipid metabolism in the oleaginous alga *Cyclotella cryptica* suggested the presence of a more efficient system to process pyruvate, and a higher number of enzymes for triacylglycerol synthesis, which would be responsible for the species’ high productivity [22]. Among the symbiotic marine dinoflagellates, which represent attractive sources of secondary metabolites, *Symbiodinium* is known to produce the polyketide symbioimine that suppresses differentiation into osteoclasts, and thus would be a candidate for treating osteoporosis [27]. Enzymatic pathways involved in the synthesis of polyketides have been studied as well. For example, *Symbiodinium* genomes have been compared to study polyketide synthases (PKS, modular enzyme systems producing important products such as antibiotics) and non-ribosomal peptide synthases (NRPS, enzymes producing non-ribosomal peptides, often with pharmacological properties), with results showing that the former are more diversified than the latter, and revealing which evolutionary processes have contributed to this diversification [28]. 

Comparative genomics can aid in the identification of the genetics basis of desirable traits, and knowledge of the genes responsible for a given phenotype enables the use of genetic engineering to improve growth, productivity and/or yield of specific molecules (see the genetic engineering paragraph below). Genome mining in prokaryotes is a routine approach to discover novel natural products [29]. However, this is not yet the case for unicellular microalgae. Homology-based searches can be conducted with BLAST (Basic Local Alignment Search Tool) [30] and HMMER [31]. Alternatively, when the traditional sequence search methods fail, novel approaches for the identification of genes and/or proteins, for which no orthologs have yet been studied, can be applied. For example, Liu (2017) [32] proposed a machine learning algorithm, using artificial recurrent neural networks (RNN), as an option for classification of protein functions directly from the primary sequence without sequence alignment. The tool permits the detection of distant homologies, allowing the discovery of proteins that are evolutionarily associated [32]. Many of the genome mining software packages developed for prokaryotes are designed to discover Biosynthetic Gene Clusters (BGC). While in eukaryotes, enzymes for a given metabolic pathway are generally distributed at different genomic locations, it is worth reporting that the recently discovered genes for the biosynthesis of the microalgal toxin domoic acid (DA) are arranged in a cluster in the genome of two DA producing species, the diatoms *P. multiseries* and *P. multistriata* [33]. We tried to look for DA biosynthetic genes loading the genome of *P. multistriata* in the fungal version of antiSMASH, and the tool did not predict any of them.

Even more challenging for MNPs discovery, marine metagenomics is enlarging the amount of sequence data to mine in search of pathways of interest. Metagenomics datasets can be found through portals such as JGI IMG/M (Integrated Microbial Genomes and Microbiomes) and GOLD (Genomes OnLine Database) (Table 2) [34,35]. Particularly promising is the recent creation of the Ocean Gene Atlas (http://tara-oceans.mio.osupytheas.fr/ocean-gene-atlas/), deployed with the Ocean Microbial Reference Gene Catalog (OM-RGC), comprising mostly prokaryotic gene sequences associated with both Tara Oceans and Global Ocean Sampling (GOS) (https://www.ncbi.nlm.nih.gov/books/NBK6855/), and the Marine Atlas of Tara Ocean Unigenes (MATOU) composed of >116 million eukaryote unigenes [36].

### 2.2. Transcriptomics

While available marine microalgal genomes are few, several transcriptomes have been sequenced. Numerous transcriptomes have been carefully annotated and studied, providing an unprecedented view into the diverse transcriptional programs operating inside these organisms. Transcriptome sequences of marine microalgae have been deposited in public databases (e.g., GenBank), and generally, both raw reads and assembled transcripts are available. A specific web-based database, named AlgaePath, is available for the freshwater green algae *Chlamydomonas reinhardtii* and *Neodesmus* sp. UTEX 2219-4 (http://algaepath.itps.ncku.edu.tw/; [58]). AlgaePath integrates gene information, biological pathways and NGS datasets for these two species.

In addition to single transcriptome sequencing projects [59,60,61,62,63], the Marine Microbial Eukaryotic Transcriptome Sequencing Project (MMETSP) provided more than 650 functionally-annotated, assembled, and publicly available transcriptomes of the most ecologically abundant and significant marine eukaryotes [1,64] (Table 2).

Several transcriptome studies are available for microalgae, responsible for harmful algal blooms. Examples are the dinoflagellates *Alexandrium catenella* [65], *Amphidinium carterae* [61], *Azadinium spinosum* [66] and *Karenia brevis* [67], but also the toxin producing diatom *P. multistriata* [68] and *P. multiseries* [33]. Other transcriptome sequencing projects focused on non-toxic microalgal species known to produce high amounts of bioactive compounds, such as lipids for cosmeceutical applications or biofuel production [69].

Considering the great market interest of MNPs, the transcriptomic approach has been used in order to reveal the gene pathways responsible for their synthesis. An example is the case of *A. catenella* [65]. Considering that little is known about the biosynthetic genes responsible for toxin production in dinoflagellates, Zhang et al. [65] compared transcriptome profiles of a toxin-producing dinoflagellate, *A. catenella,* and its non-toxic mutant form. 101 putative homologs of 12 cyanobacterial *sxt* genes (genes involved in the synthesis of saxitoxin and its derivatives) were identified. Among the down-regulated genes in the non-toxic mutant, expression of the transcript assigned to *sxtA*, the initiator of toxin biosynthesis in cyanobacteria, was significantly down-regulated, suggesting that it might be directly involved in toxin biosynthesis. In addition, the authors identified *sxtO* and *sxtZ* genes in dinoflagellates for the first time. 

Similarly, Brunson et al. [33] studied domoic acid (DA) biosynthetic genes, by using growth conditions known to induce DA production in *P. multiseries*. They suggested that the gene candidates for DA biosynthetic (Dab) enzymes are dabA (terpene cyclase), dabB (hypothetical protein), dabC [a-ketoglutarate (aKG)–dependent dioxygenase], and dabD (cytochrome P450, CYP450). 

Other transcriptomic studies focused on the study of PKSs responsible for the synthesis of toxins and other polyketides with interesting biotechnological applications (e.g., anticancer and antifungal activity and/or beneficial effects for the treatment of Alzheimer’s disease [70,71,72,73,74]). 

Finally, another approach exploited transcriptome sequences to find transcripts coding for enzymes that are used for medical applications. This is the case of the enzyme L-asparaginase (EC 3.5.1.1), an enzyme that catalyzes the hydrolysis of L-asparagine to L-aspartic acid. L-asparaginase is used to treat acute lymphoblastic leukemia, acute myeloid leukemia, and non-Hodgkin’s lymphoma. Cancer cells have a reduced capacity to produce asparagine synthetase, and rely on asparagine supplied directly from the blood. By using L-asparaginase, the supply of asparagine is reduced and the growth of cancer cells is inhibited. A transcript for this enzyme was found in the transcriptome of the dinoflagellate *A. carterae* [61].

### 2.3. Proteomics

Genomics and transcriptomics alone are insufficient to understand the complex biology of microalgae, and should be complemented with a proteomic approach. Proteomics explores the mechanisms involved in many biological processes and network functions by providing information on proteins, including post-translational modification, subcellular localization and protein-protein interaction. The first proteomic analysis in microalgae was conducted in the unicellular freshwater green alga *C. reinhardtii,* chosen as a model organism. 

However, *Chlamydomonas* alone certainly is not representative of the physiology of all species of biotechnological interest. In the last decade, proteomics approaches have been mostly focused on the model diatom *P. tricornutum* and on oleaginous microalgae, among others *Chlorella vulgaris* and *Fistulifera solaris,* species favorable, in particular, for biofuel and biodiesel production (see as example: [39,75,76,77,78]). One example is the case of Siegler and collaborators who, using a semi-quantitative proteomics approach, investigated the composition of the lipid bodies (LBs) in the oleaginous microalga *Lobosphaera incisa* [79]. Similarly, Davidi et al. [80] performed a proteomics analysis of the lipid droplets organelles in the green microalgae *Dunaliella bardawil* and *D. salina.* Their analysis revealed the accumulation of two different types of lipids, cytoplasmic lipid droplets (CLD) and β-carotene-rich (βC) plastoglobuli. In particular, the high nutritional and pharmacological value of β-carotene for humans has promoted intensive research aimed to clarify its biosynthesis and regulation [80]. Other studies focused mostly on a proteomic analysis of oleaginous microalgae in the nitrogen-deprived growth condition that tends to increase lipid production. Examples are the cases reported by Longworth et al. [81] who examined, in the model diatom *P. tricornutum*, the proteome response of lipid accumulation induced during nitrogen depletion [81], and by Garibay-Hernández and collaborators who performed the first membrane proteome of the non-sequenced microalga *Ettlia oleoabundan*s from nitrogen-deprived cultures [82]. 

Rai and colleagues investigated the proteome dynamics of *Chlorella* sp. FC2 IITG in nitrogen starvation, using two high-throughput complementary proteomics platforms: DIGE (2D differential in-gel electrophoresis) and iTRAQ (isobaric tagging for relative and absolute quantitation), two methods widely used in ecotoxicology studies. In particular, they found an up-regulation of hydroxyacyl-ACP dehydrogenase and enoyl-ACP reductase known to be involved in lipid accumulation [77]. However, the proteomics analysis of these microalgal species is still limited, since these organisms have a high lipid body protein (LBPs) content, and protein extraction is a difficult and very time-consuming task [83]. Very recently, Fernández-Acero and colleagues [84] reported the first whole proteomic description under industrial conditions of the microalgae *Nannochloropsis gaditana,* recognized as a promising species for the production of different bioactive compounds with biotechnological applications. They identified a total of 655 proteins enlarging the proteome database Uniprot. Two proteins with potential applications were characterized in detail, a prohibitin (GenBank: XM_005852605.1) involved in cellular replication control, and a resistance to phytophthora protein (GenBank: XM_005854224.1), capable of conferring resistance to the oomycetes *Phytophthora infestans* [84]. In order to manage the proteomic data generated so far, different existing databases, such as Protein Data Bank archive [85] and Uniprot [86], have been enlarged to include algae proteins. In addition, new databases have been developed. Example of user-friendly open access and comprehensive database with specific algal proteome information is Algal Protein Annotation Suite (Alga-PrAS), a comprehensive database with physicochemical, structural and functional information [87] (Table 2).

### 2.4. Metabolomics

Metabolites are dynamic, and their properties and levels of biosynthesis depend on genetic and/or environmental changes. Their comprehensive analysis is defined as metabolomics. Marine organisms produce an extraordinary variety of often unique active secondary metabolites that differ from those identified in terrestrial organisms, because they have special metabolic patterns closely linked to the unique features of their environments, consisting for example of continuous variations in light, pressure, nutrient, salinity and temperature. Bioactive secondary metabolites from microalgae are referred to as High-Value Molecules (HVM) [88], and are of major interest for drug discovery (e.g., [89]). Their synthesis can be triggered by physiological and/or environmental stimuli (e.g., the presence of predators, nutrient depletion or starvation, light, as found in [90,91]). To date many bioactive metabolites (carotenoids, polyunsaturated fatty acids (PUFAs), polysaccharides, glycolipids), have been identified in various marine microalgae such as diatoms, flagellates, dinoflagellates, and other species [88,92]. 

The desired metabolites are identified and analyzed with a targeted approach, and generally, the number of metabolites isolated/obtained is low. To perform high throughput metabolite profiling, an untargeted approach is required. Nuclear magnetic resonance (NMR) and mass spectrometry (MS)-based techniques are among the most popular technologies available [93]. However again, there are few examples of metabolomic profiles from microalgae reported in the literature, and research in this area is still in its infancy.

As for proteomics, most metabolomics studies are focused on oleaginous marine microalgae. For example, Willette and colleagues reported the metabolomic and lipidomic profiles of *Nannochloropsis salina* under cold stress, and observed a total fatty acids accumulation [94]. The most interesting step forward in this area of research has been recently accomplished by Sun and colleagues [95]. They developed a method which allows direct extraction of metabolites from single living *Scrippsiella trochoidea* cells for MS analysis, called Single-probe MS technology. The cells of *S. trochoidea*, a non-toxic marine dinoflagellate, were compared in the context of diurnal light changes. The technique has the potential to allow metabolomic analysis of individual phytoplankton cells, opening the door to targeted analyses that minimize cell manipulation, and preserving metabolic variability at the cellular level [95]. Different open-access databases have focused on animal, plant and microbe (including bacteria, archaea and fungi) metabolites that have been developed to assign structures to spectral peaks observed in metabolomics experiments [96]. By comparison, metabolomics in microalgae is a late-comer, and there are no dedicated databases. Examples of publicly-accessible databases containing cross-species metabolomics information and extensive links to other public databases, such as the Kyoto Encyclopedia of Genes and Genomes (KEGG) [97] and PubChem [98], are listed in Table 2. These include MetaCyc, which describes experimentally-studied metabolic pathways from all domains of life, microalgae included [99].

## 3. Genetic Engineering to Increase High-Value Product Production

Genetic engineering is extensively used to improve different properties of plants, mainly crops. For several microalgae identified as potential producers of valuable compounds, the possibility of manipulating genes is an attractive opportunity, and could lead to leaps forward in reducing costs and improving production efficiency. Advanced methods for gene manipulations are available for only a handful of species: for biotechnological applications, well-developed systems are green microalgae, such as *Chlamydomonas* and *Chlorella* species, while for marine species the two most used systems are the two stramenopiles *Nannochloropsis* and *P. tricornutum* [78]. Factors that have limited the development of genetic toolkits in marine microalgae until now include the presence of a rigid cell wall for many groups, which makes transformation methods inefficient, and the scarcity of suitable markers for selection and promoters [100,101]. Moreover, gene targeting via homologous recombination has been reported for *Nannochloropsis* [102] and for the picoalga *Ostreococcus tauri* [103], but not for diatoms and dinoflagellates.

Recent initiatives to expand the number of marine species amenable to manipulations (MMI, https://www.moore.org/article-detail?newsUrlName=doubling-down-on-developing-genetic-tools-for-marine-microbial-ecology) and the development of the CRISPR/Cas9 system [104], are bringing about major changes in the field, and are expected to greatly enhance our ability to engineer genomes of species deemed promising for natural products production. Research is advancing rapidly, especially for diatoms. An overview of the tractable species and of the methods developed are provided by [105,106]. In this field, engineered microalgae can be useful in two ways: (i) they can be modified to enhance growth properties, photosynthetic efficiency or the production of given molecules; (ii) they can be used as expression systems, as an alternative to bacterial or yeast expression systems, or to produce compounds of interest with a better efficiency.

Since microalgae have been regarded as promising systems to produce biofuels since the 1980s [107], research in this field has proliferated, and most efforts have been made to engineer their lipid pathway. In addition to biofuels, the lipid pathway is a target of election also because of their high content in PUFAs, highly valuable for animal and human consumption. Decades of research are leading to the production of significant results. *P. tricornutum* transgenic strains overexpressing the Δ5-elongase from *O. tauri* accumulate the high value omega-3 long chain polyunsaturated fatty acid docosahexaenoic acid (DHA) [108], and have been shown to grow efficiently on large scales [109]. Zulu et al. [103] overexpressed a yeast diacylglycerol acyltransferase (*ScDGA1*) and a plant oleosin (*AtOLEO3*), and succeeded in obtaining transgenic strains richer in triacylglycerols [110].

Other efforts have been devoted to improve the carotenoid content in diatoms: one attempt to overexpress the phytoene synthase (PSY) in *P. tricornutum* led to increased fucoxanthin content in one transformant by approximately 1.45-fold with respect to the levels in the wild-type [111]. In another study, overexpression of the 1-deoxy-d-xylulose 5-phosphate synthase (DXS) and the PSY genes, in the same diatom species, led to a 2.4-fold and a 1.8-fold higher fucoxanthin content, respectively [112].

TALEN (transcription activator-like effector nucleases) -mediated inactivation of UDP-glucose pyrophosphorylase, a gene involved in the carbohydrate storage pathway, led to a 45-fold increase in triacylglycerol accumulation [113]. 

CRISPR/Cas9 *N. gaditana* mutants for a transcriptional regulator doubled their lipid productivity while retaining growth properties similar to those of the wild type [114].

More recently, Da-Wei Li and colleagues [115] characterized the roles of the transcription factor bZIP1 (designated NobZIP1) in *N. oceanica* through overexpression and RNAi-mediated silencing. Overexpressing cells exhibited an increase in the accumulation of PUFAs, carbohydrates and proteins. On the other side, the silenced cells showed an increase in total saturated fatty acids, and in particular, in the extracellular lipid content. The results suggested a role for NobZIP1 in regulating the expression of key genes related to lipid and carbohydrate metabolism.

As far as the possibility of using marine microalgae as expression systems, *P. tricornutum* has been tested as a system for the production of human antibodies [116] and of bioplastics [117], with successful results. In the first case, fully-assembled and functional monoclonal human IgG antibody against the Hepatitis B surface protein accumulated to almost 9% of total soluble protein, reaching values similar to those achieved in plant systems [116]. In the second case, the bacterial poly-3-hydroxybutyrate (PHB) pathway was introduced into the diatom, leading to PHB levels of up to 10.6% of algal dry weight [117]. Very recently, within an EU-funded project called TriForC, *P. tricornutum* has been engineered for high-value plant terpenoid production, by introducing three plant enzymes in the microalga (a *Lotus japonicus* oxidosqualene cyclase and a *Medicago truncatula* cytochrome P450, along with its native reductase) [118]. Triterpenes are secondary metabolites that are used by plants as natural self-defense mechanisms from pests, and that have been studied as novel bio-pesticides, or as anti-inflammatory agents. It will be now important to ensure that regulations are in place to allow an environmentally safe growth of GMO strains in outdoor systems [119].

## 4. European Projects and Infrastructures

Despite the number of compounds isolated from marine organisms, marine biotechnology projects focusing on drug discovery from marine microalgae have been very scarce in the 7th Framework Program (FP7) and successive Horizon 2020 (H2020) program [120]. These programs funded projects for implementing sampling and cultivation technologies, to sequence genomes and transcriptomes, and to study the proteome and metabolome, in order to speed up the drug discovery pipeline. Under these programs, projects focusing on microalgae are FP7-funded projects BAMMBO, GIAVAP, PharmaSea, SUNBIOPATH, and currently in progress, H2020 projects such as EMBRIC, NoMorFilm and TriForC (see Table 3 for project details). Of these, only GIAVAP (focusing on two freshwater algae, *Parietochloris incisa* and *Haematococcus pluvialis*) and PharmaSea made use of -omics approaches.

PharmaSea aimed to find new products for development in three accessible market sectors, health (infection, inflammation, neurodegenerative diseases), personal care and nutrition, from microalgae and bacteria. Results on microalgae reported that three diatom species, i.e., *Cylindrotheca closterium*, *Odontella mobiliensis* and *Pseudo-nitzschia pseudodelicatissima*, displayed specific anti-inflammatory activity, the diatom *Skeletonema marinoi* showed anticancer activity (blocking human melanoma cell proliferation), while the diatoms *Leptocylindrus aporus* and *Leptocylindrus danicus* exhibited anti-biofilm activity against the bacteria *Staphylococcus epidermidis* [14]. The chemical nature of these bioactive compounds is currently under investigation. Transcriptome sequencing of bioactive species has been performed, or is in progress, in order to identify the enzymes involved in the synthesis of metabolites with possible pharmaceutical applications (e.g., [61,63]). 

In addition, the European Marine Biological Research Infrastructure Cluster (EMBRIC) focuses on promoting drug discovery from marine organisms by building inter-connectivity between science, industry and regions (https://cordis.europa.eu/project/rcn/198465/factsheet/en). It aims to form a permanent cluster of research institutes (RIs), which will foster innovation in marine biotechnology [121]. Unpublished results indicate that microalgal strains with active anti-proliferative activity have been found, and further characterization for the purpose of identifying the molecules responsible for this bioactivity is currently underway. EMBRIC also explored the possibility to apply metabolic engineering to marine microalgae, with successful results for the enhancement of carotenoids in the picoalga *O. tauri*, and work devoted to interfere with transcription factors or signaling in diatoms (see www.embric.eu).

Another major target pathway is the carotenoid pathway. Theoretically, productivities of PUFA or carotenoids such as astaxanthin, fucoxanthin or lutein, can be doubled or tripled by using multiple subsequent genetic modification steps. Data in this context have been obtained in the project GIAVAP, which had the aim to genetically modulate and successfully cultivate a wide variety of microalgae (see project https://cordis.europa.eu/result/rcn/155764_en.html). The pan-European research infrastructure EMBRC (The European Marine Biological Resource Centre), dedicated to marine biology and ecology research, also offers access to -omics technology platforms and data analysis resources (www.embrc.eu). Under the direction of EMBRC, the consortium ASSEMBLE Plus (Association of European Marine Biological Laboratories Expanded), constituted of partners from 16 different countries, operates by providing access to resources and facilities, and supporting exchange between scientists from public and private institutions (http://www.assembleplus.eu/about).

The intergovernmental infrastructure ELIXIR (https://www.elixir-europe.org/) operates in order to deal with the increasing amount and complexity of data. It integrates and manages the bioinformatics resources (e.g., databases, software tools, training materials, cloud storage and supercomputers) available for the marine field being generated by publicly-funded research across Europe.

## 5. Conclusions and Future Perspectives

During recent years, -omics resources provided novel opportunities to identify and characterize high-value bioactive compounds derived from marine microalgae. However, a user that ventures into the discovery of MNPs from marine microalgae, faces a fragmentation of data sources, incomplete reference datasets, and a lack of dedicated software. Despite the decreasing costs of the -omics technologies, the datasets available are still too few for comparative tools to work effectively, and more investments into the production of data from microalgal species is desirable. Moreover, the vastness of large-scale -omics datasets requires the integration of these data under bioinformatics models/tools. These tools are already available in the field of human diseases and of plants [122,123]. To date, the solely integrated approach applied in algae is reported by Maes and colleagues [124]. The authors published an integrated pipeline, called MinOmics (Methods for Integrated analysis of Multiple Omics datasets) to manage various biological data, such as genes, transcripts and proteins, deriving from the freshwater microalga *C. reinhardtii* [117]. Improvements in this field can be made by extending this tool to other -omics data generated from other microalgae, especially those of ecological and biotechnological interest. Machine Learning (ML), and in particular Deep Learning (DL), two subfields of artificial intelligence, are able to handle unstructured big data sets, and find the correlation among them. They have already been successful in several biology areas [125,126] and could also be helpful to correlate the available microalgal -omics datasets for MNPs discovery. Combination of these -omics resources and physiological data will identify the bioactive species, as well as the culturing and environmental conditions in which higher amounts of the metabolite of interest are produced. Hence, multi-disciplinarity will finally speed up drug discovery from marine species.

## Figures and Tables

**Table 1:** Marine microalgal genomes available.

Available Genomes	Description	References
*Aureococcus anophagefferens*	Ochrophyta	[37]
*Bathycoccus prasinos*	Chlorophyta	[38]
*Chlorella vulgaris UTEX395*	Chlorophyta	[39]
*Coccomyxa sp. C-169*	Chlorophyta	[40]
*Dunaliella salina*	Chlorophyta	[41]
*Emiliania huxleyi*	Haptophyta	[42]
*Fistulifera solaris*	Bacillariophyta	[21]
*Fragilariopsis cylindrus*	Bacillariophyta	[43]
*Guillardia theta*	Cryptophyta	[44]
*Micromonas pusilla (CCMP1545)*	Chlorophyta	[45]
*Micromonas pusilla (NOUM17)*	Chlorophyta	[45]
*Micromonas sp. RCC299*	Chlorophyta	[45]
*Nannochloropsis gaditana CCMP1894*	Eustigmatophyte	[46]
*Nannochloropsis gaditana*	Eustigmatophyte	[47]
*Ostreococcus lucimarinus*	Chlorophyta	[48]
*Ostreococcus sp. RCC809*	Chlorophyta	https://genome.jgi.doe.gov/OstRCC809_1/OstRCC809_1.home.html
*Ostreococcus tauri*	Chlorophyta	[49,50,51]
*Phaeodactylum tricornutum*	Bacillariophyta	[52]
*Picochlorum costavermella*	Chlorophyta	[53]
*Pseudo-nitzschia multiseries*	Bacillariophyta	https://genome.jgi.doe.gov/Psemu1/Psemu1.home.html
*Pseudo-nitzschia multistriata*	Bacillariophyta	[23]
*Skeletonema costatum*	Bacillariophyta	[24]
*Symbiodinium kawagutii*	Dinoflagellata	[54]
*Symbiodinium microadriaticum*	Dinoflagellata	[55]
*Thalassiosira oceanica*	Bacillariophyta	[56]
*Thalassiosira pseudonana*	Bacillariophyta	[57]

**Table 2:** Links to databases and websites in which marine -omics data can be found.

**Genomics**	Pico-Plaza	https://bioinformatics.psb.ugent.be/plaza/versions/pico-plaza/
	Joint Genome Institute (JGI) Genome Portal for Algae	https://genome.jgi.doe.gov/algae/algae.info.html
	Ensembl Protists	https://protists.ensembl.org/species.html
	BioCyc	https://biocyc.org/
	PlaNet	http://www.gene2function.de/
	DiatomCyc	http://www.diatomcyc.org/
**Meta-Genomics**	iMicrobe	www.imicrobe.us
	Ocean Gene Atlas	http://tara-oceans.mio.osupytheas.fr/ocean-gene-atlas/
	JGI Integrated Microbial Genomes	https://img.jgi.doe.gov/cgi-bin/m/main.cgi/
	GOLD	www.genomesonline.org/cgi-bin/GOLD/index.cgi
**Transcriptomics**	MMETSP	https://www.imicrobe.us/#/projects/104
	AlgaePath	http://algaepath.itps.ncku.edu.tw/
	Alganaut	https://alganaut.uts.edu.au/Clusterview/
**Proteomics**	Alga-PrAS	http://alga-pras.riken.jp/
	Uniprot	https://www.uniprot.org/
	Protein Data Bank archive (PDB)	www.rcsb.org
**Metabolomics**	ChEBI	www.ebi.ac.uk/chebi/
	DrugBank	www.drugbank.ca/
	PubChem	http://pubchem.ncbi.nlm.nih.gov
	KEGG Compound	https://www.genome.jp/kegg/compound//
	MetaCyc	https://metacyc.org/
	MMCD	http://mmcd.nmrfam.wisc.edu/
	MetaboLights	https://www.ebi.ac.uk/metabolights/

**Table 3:** The table reports the starting and end dates and the relative websites of the EU projects discussed in this review.

Project Name	Starting Date	Ending Date	Website
BAMMBO	01/03/2011	01/03/2014	www.bammbo.eu, http://cordis.europa.eu/project/rcn/97837_en.html
GIAVAP	01/01/2011	31/12/2013	http://cordis.europa.eu/project/rcn/97420_en.html
PharmaSea	October 2012	March 2017	www.pharma-sea.eu
SUNBIOPATH	01/01/2010	28/02/2013	http://cordis.europa.eu/project/rcn/92954_en.html
EMBRIC	01/06/2015	31/05/2019	www.embric.eu
NoMorFilm	01/04/2015	31/03/2019	www.nomorfilm.eu
TriForC	01/10/2013	30/09/2017	https://cordis.europa.eu/project/rcn/110952/factsheet/en
